# Methods for comparative metagenomics

**DOI:** 10.1186/1471-2105-10-S1-S12

**Published:** 2009-01-30

**Authors:** Daniel H Huson, Daniel C Richter, Suparna Mitra, Alexander F Auch, Stephan C Schuster

**Affiliations:** 1 Center for Bioinformatics ZBIT, Tübingen University, Sand 14, 72076 Tübingen, Germany; 2 310 Wartik Laboratories, PennState University, Center for Comparative Genomics, Center for Infectious Disease Dynamics, University Park, PA 1803, USA

## Abstract

**Background:**

Metagenomics is a rapidly growing field of research that aims at studying uncultured organisms to understand the true diversity of microbes, their functions, cooperation and evolution, in environments such as soil, water, ancient remains of animals, or the digestive system of animals and humans. The recent development of ultra-high throughput sequencing technologies, which do not require cloning or PCR amplification, and can produce huge numbers of DNA reads at an affordable cost, has boosted the number and scope of metagenomic sequencing projects. Increasingly, there is a need for new ways of comparing multiple metagenomics datasets, and for fast and user-friendly implementations of such approaches.

**Results:**

This paper introduces a number of new methods for interactively exploring, analyzing and comparing multiple metagenomic datasets, which will be made freely available in a new, comparative version 2.0 of the stand-alone metagenome analysis tool MEGAN.

**Conclusion:**

There is a great need for powerful and user-friendly tools for comparative analysis of metagenomic data and MEGAN 2.0 will help to fill this gap.

## Background

Metagenomics is a rapidly growing field of research that aims at studying uncultured organisms to understand the true diversity of microbes, their functions, cooperation and evolution, in environments such as soil, water, ancient remains of animals, or the digestive system of animals and humans. Although it is clear that communities of microbes play a vital role in such systems, a more detailed understanding is only beginning to emerge. A main promise of metagenomics is that it will accelerate drug discovery and biotechnology by providing new genes with novel functions.

Currently, the key approach used in metagenomics is large-scale sequencing of environmental samples. The recent development of ultra-high throughput sequencing technologies [1,2], which do not require cloning or PCR amplification, and can produce huge numbers of DNA reads at an affordable cost, has boosted the number and scope of metagenomic sequencing projects, see [3,4]. The analysis of such datasets is aimed at determining and comparing the biological diversity and the functional activity of different microbial communities.

Computationally, species identification relies on the use of reference databases or reference phylogenies that contain of sequences of known origin and gene function. The most prominently used databases are the NR and NT databases [5]. Unfortunately, substantial database biases toward model organisms present a major hurdle for metagenomic analysis, and in a typical metagenome dataset as much as 90% of the reads may exhibit no similarity to any known sequence. However, this problem is beyond the scope of this paper. Early 2007, our group released and published the first publicly available, stand-alone analysis tool for metagenomic data, called MEGAN [6,7]. We initially developed this tool to analyze the microbial community present in a sample of mammoth bone [8]. MEGAN takes as input the result of a BLAST [9] comparison of a set of metagenomic reads against one or more reference databases and produces as output a taxonomical analysis of the sample, obtained by assigning the reads to different nodes in the NCBI taxonomy using an "LCA-algorithm".

As an exploration tool designed and optimized to run on a laptop, MEGAN complements other systems and resources for metagenome analysis, which are offered in the form of databases, web portals and web services, such as [10-14].

MEGAN now has over 400 registered users working in many different biological labs around the world. It is routinely used at the Joint-Genome-Institute (JGI) both in quality control and also to provide initial analyses of newly sequenced datasets. Other users include researchers at the J.C. Venter Institute studying viral populations. In a recent publication [15], we demonstrate how to use the software for meta-transcriptomics, as well.

Increasingly, the emphasize of metagenome analysis is shifting from species and functional identification for individual datasets toward comparative analysis. This paper addresses the latter issue and provides solutions to questions such as: Given two or more metagenome datasets, how similar or different are their taxonomical and functional profiles? Are observed differences statistically significant? Have enough reads been sequenced, i.e. what is the current "rate of discovery" as a function of the number of reads sequenced? In the following section, we will discuss some new ideas for analyzing individual metagenome datasets. Then, we will focus on new comparative methods. Finally, we will illustrate the application of the methods in two comparisons, one comparing the contents of a human gut [16] with the contents of a mouse gut [17] and the other comparing a soil sample [18] with a recent marine sample [19].

The ideas presented in this paper are all quite simple and unsophisticated. The main merit of this work lies in the integrated implementation of the methods in the form of a very robust and user-friendly program, which is easily used by biologists. The implementation goes well beyond the hastily written "proof of concept" implementations that so often accompany method papers. We are currently beta-testing version 2.0 of the MEGAN software, which implements all ideas presented in this paper. The latest beta version can be obtained from our website at [20].

## Methods

One goal of metagenome analysis is to determine the taxonomical content of a dataset [6,21]. There are two main approaches toward doing this.

The *phylogenetic approach *is based on carefully chosen genes that are believed to provide robust phylogenetic information [22,23], see [21,24]. When randomly-targeted sequencing is used, only a small fraction of the sequences will correspond to such phylogenetic markers [21,25]. Often, universal primers are employed to specifically target the phylogenetic markers. The DNA sequences obtained are usually aligned into precomputed reference alignments and placed into precomputed reference trees, using fast heuristics and then taxonomical placements are deduced from this.

The *taxonomical approach *places reads directly into the NCBI taxonomy, based on the similarity of the reads to sequences in one or more reference databases. As randomly sequenced reads will exhibit very different levels of evolutionary conservation, it is important to make use of all ranks of the NCBI taxonomy, placing more conserved sequences higher up in the taxonomy (i.e. closer to the root) and more distinct sequence onto nodes that are more specific (i.e. closer to the leaves, which represent species and strains). This can be done using the *LCA algorithm *and is the basis of the MEGAN program.

In summary, the LCA algorithm works as follows. A sequencing read is compared against a database of reference sequences, such as the NCBI NR database, and the taxon information of significant matches is extracted and mapped onto the leaves of the NCBI taxonomy. The leaves of the NCBI taxonomy represent different species and strains. The LCA algorithm computes the lowest common ancestor of all these hits, which will correspond to some higher-order taxon, and will then assign the read to that taxon. In this way, species-specific sequences will be assigned to the leaves or specific taxa, whereas sequences that are conserved among different species, or that are susceptible to horizontal gene transfer, will be assigned to taxa of less-specific rank. See the original paper [6] for more details.

Both approaches have different advantages and draw-backs. The phylogenetic approach can use established phylogenies that are well understood and targeted sequencing provides much more informative data per sequencing run. However, a commonly acknowledged draw-back is that the "universal primers" employed may produce only a subset of the true spectrum of different sequences. On the other hand, random sequencing is often used in metagenomics to analyze the gene content of a community and then the taxonomical approach can make full use of the data and can be complemented by a phylogenetic approach.

### Rate of discovery

**Figure 1:**
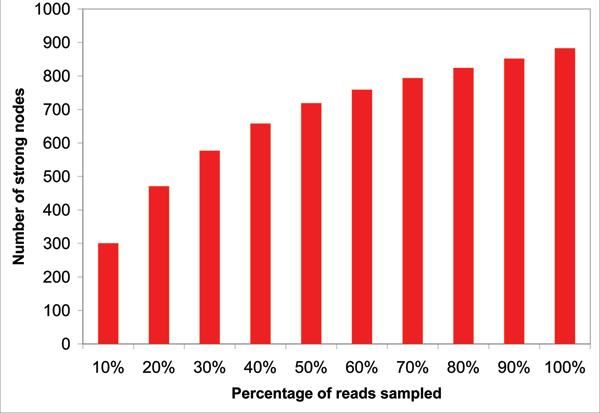
A discovery rate plot computed by MEGAN 2.0 for the mouse gut dataset. The *x*-axis represents the percentage of reads subsampled from the total dataset and the *y*-axis represents the number of strong nodes (with *t *= 5) computed by the LCA algorithm, approximating the number of identified species. The datapoint at 10 × *t *% is based on *t *independent runs.

One important question is whether the level of sequencing performed for a given sample is sufficient to capture the most abundant taxa. This can be addressed by plotting the *discovery rate *of a dataset, which is obtained by repeatedly selecting random subsamples of the dataset at 10, 20 ..., 90% of the original size, and then plotting the number of taxa predicted by the LSA algorithm, see Figure 1. This graph can be used to estimate (roughly) how many additional species are likely to be discovered if one were to increase the number of reads by a factor of two, say.

In this, to estimate the number of species, one might first consider counting the number of leaves of the taxonomy to which reads have been assigned. However, this number may be confounded by the presence of different strains and isolates. To avoid this problem, in our implementation in MEGAN 2.0 we use the number of *strongly supported *nodes as a proxy for the number of species. We say that a node *v *in the NCBI taxonomy is *strongly supported at level t*, where *t *is a small number (≈ 5), if *v *has been assigned *t *or more reads and no node below *v *has that property.

### Functional assessment

In a functional analysis, the goal is to determine which types of genes are available at what relative levels of abundance. Such an analysis can be based on sequences obtained by random sequencing either of the genomic DNA in a metagenome, or (reverse transcribed) RNA. In the former case, the coding potential is analyzed, whereas in the latter case, the focus is on gene expression. A general strategy is to compare the reads against reference databases of gene sequences such as COG [26] and SEED [11].

**Figure 2:**
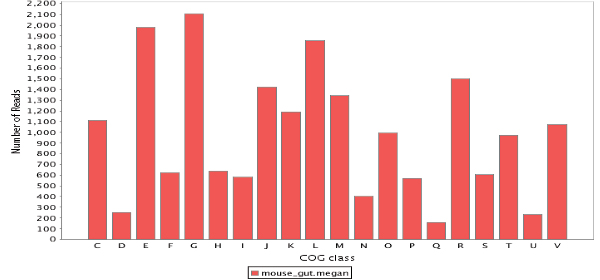
A classification of all COGs determined in the mouse gut sample.

A number of sequences available in the NR database are annotated by COG [26] identifiers. Hence, after BLAST comparison of a metagenomic dataset with the NR database, a first analysis of the types of genes present in the dataset can be performed by extracting all COG identifiers from the BLAST hits and then summarizing the relative abundances of the different COG categories, see Figure 2.

### Meta-data analysis

**Figure 3:**
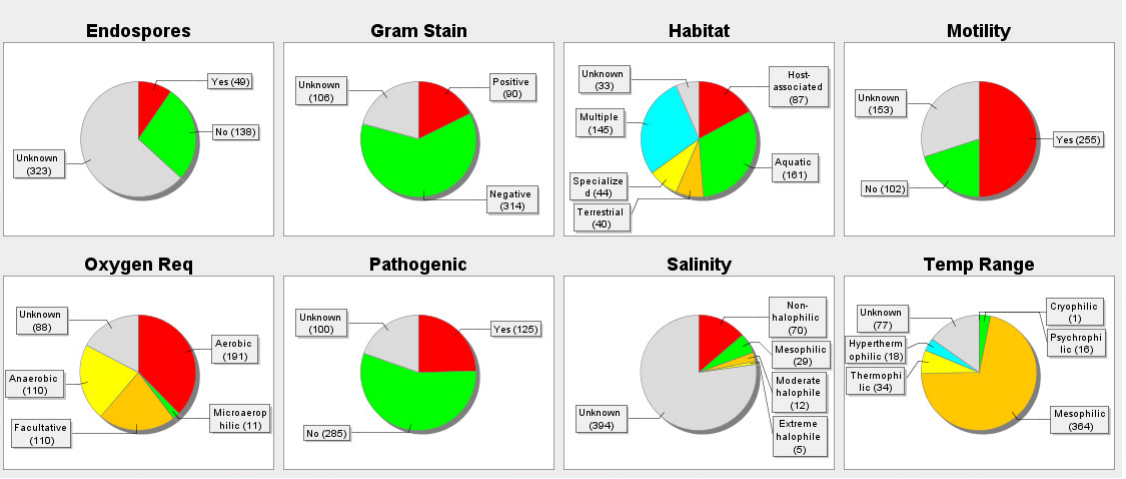
Summary of the microbial attributes of the soil dataset based on the NCBI's "Prokaryotic Attributes Table". In each pie chart, the number of classified species having the indicated property is displayed.

The result of a taxonomical analysis can be enhanced by using "meta-data" to summarize the identified species. For example, the "Prokaryotic Attributes Table" (obtainable from the NCBI website) lists attributes of microbes that describe their cellular features, environment, temperature, pathogenicity and relevance for diseases. A summary of an analysis based on such attributes is shown in Figure 3.

### Taxonomy-guided capture of reads

Once a first analysis has been performed and reads have been assigned to taxa, it is often desirable to be able to identify and capture all reads that have been assigned to one part of the NCBI taxonomy, not only to a specific species, but also to a class, genus or other rank of the taxonomy. This is very useful, for example, when performing additional analysis such as determining the GC-content for a collection of taxa, or for sequence assembly purposes.

### Comparative visualization

**Figure 4:**
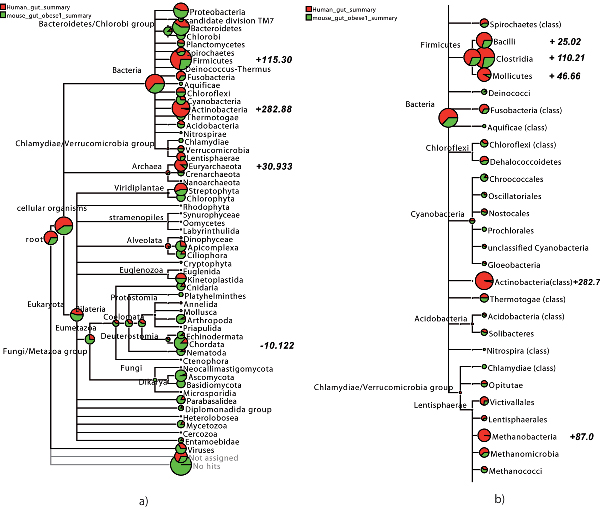
Two multiple-comparative tree views of a human gut metagenome [16] shown in red and a mouse gut metagenome [17] shown in green, as computed by MEGAN 2.0, using normalized counts. In (a), we show an overview of the taxonomy down to the phylum level, whereas in (b) we display a part of a class-level analysis. In bold we show the support values as listed in Table 1.

**Figure 5:**
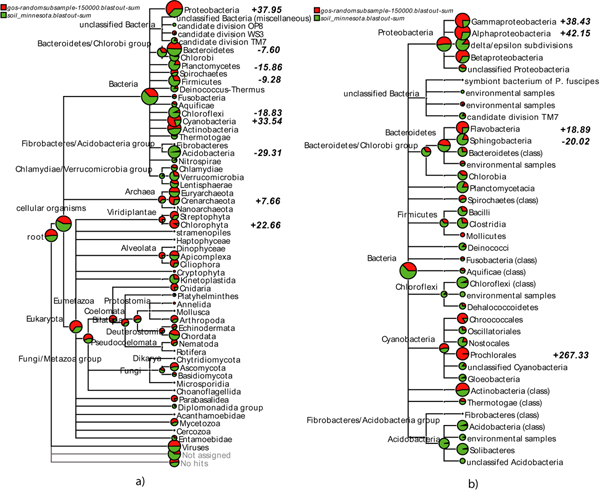
A multiple-comparative tree view of a soil metagenome [18] shown in green and a marine metagenome [19] shown in red, as computed by MEGAN 2.0. In (a), we show an overview of the taxonomy down to the phylum level, whereas in (b) we display a part of a class-level analysis. In bold we show the support values as listed in Table 2.

In a comparative analysis, different datasets are brought together and compared for taxonomical and functional content. To compare multiple datasets, we define a new *multiple-comparison tree view *in which an arbitrary number of different datasets are displayed together on a subtree of the NCBI taxonomy, as shown in Figures 4 and 5. In such a view, each node in the NCBI taxonomy is shown as a pie chart indicating the number of reads (normalized, if desired) from each dataset that have been assigned to that node. An important feature is the ability to interactively collapse or expand the presented tree at different levels of the taxonomy, so as to be able to start at a high-level view and then to drill down to a low-level comparison.

**Figure 6:**
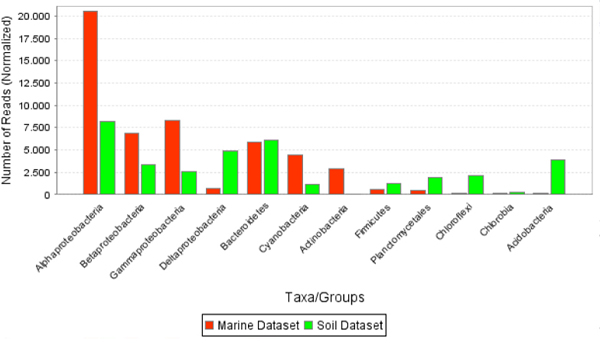
A summary of the comparison of the marine (red) and soil (green) datasets, generated at different taxonomical ranks.

For publication purposes, the ability to interactively setup and generate different types of summaries using bar and pie charts, and also heat maps for many-way comparisons, are important. We are developing an interactive and fully customizable chart viewer for MEGAN 2.0 that allows one to extract a number of different comparisons directly from the multiple comparison tree view. For example, one can generate a bar chart summarizing the number of reads assigned at any desired rank of the NCBI taxonomy, see Figure 6.

### Statistical significance

Comparative visualizations are useful to obtain an impression of how two datasets differ. For a more detailed analysis, one requires information on the statistical significance of observed differences, see Table 1 and 2. To this end, we have adapted a test developed for comparing curated subsystems in metagenomic data [27]. This test uses bootstrapping to determine for which subsystems a difference in counts is significant. This can be extended by defining a support value as the proportion of deviation given by $\frac{2\left|M-P_{50}\right|}{P_{95}-P_{5}}$, based on the average difference *M *of pairs of values sampled from the two different datasets and the percentile values *P*_*x *_obtained by resampling from the *same *dataset. In MEGAN 2.0, it will be possible to apply this test to any level of the NCBI taxonomy.

**Table 1:** Significant differences in the comparison of human gut and mouse gut metagenomes. The five most statistically significant differences in numbers of reads assigned to taxon classes in the comparison of a human gut [16] and obese mouse gut [17] metagenomes. A positive support (proportion of deviation) indicates that the difference is in favor of the human gut dataset, whereas a negative sign indicates the opposite.

Comparison of human and mouse gut datasets					
Rank	1	2	3	4	5

Phylum level Support	Actinobacteria +282.88	Firmicutes +115.30	Euryarchaeota +30.93	Chordata -10.12	Ascomycota -6.96

Class level Support	Actinobacteria +282.70	Clostridia +110.21	Methanobacteria +87.0	Mollicutes +46.66	Bacilli +25.01

**Table 2:** Significant differences in the comparison of marine and soil metagenomes. The five most statistically significant differences in numbers of reads assigned to taxon classes in the comparison of marine [19] and soil [18] metagenomes. A positive support (proportion of deviation) indicates that the difference is in favor of the soil dataset, whereas a negative sign indicates the opposite.

Comparison of marine and soil datasets					
	1	2	3	4	5

Phylum level Support	Proteobacteria +37.95	Cyanobacteria +33.54	Acidobacteria -29.31	Chlorophyta +22.67	Chloroflexi -18.83

Class level Support	Prochlorales +267.33	Thermoprotei +82.36	Oligohymenophorea +52.36	Aconoidasida +50.36	Prasinophyceae +52.33

### Dealing with very large datasets

To be able to deal with ever larger, multiple datasets on a computer with a limited amount of main memory, MEGAN 2.0 can perform the analysis of any given dataset in a new *summary *mode, in which the analysis is performed on-the-fly and none of the read or match data are loaded into memory. A summary file obtained in this way describes only how many reads were assigned to each taxon, and thus the size of such a file is independent of the size of the original input dataset.

In an ongoing study, we are using a beta version of MEGAN 2.0 to analyze datasets containing a million or more reads. As another example, a BLAST file generated for the soil sequences discussed in Section is 53 GB in size and can be parsed in less than 40 minutes on a laptop. Once parsed in this way, the data can then be saved in a summary format that can be reopened in seconds.

## Results and discussion

In this section we will illustrate some of the methods described above, using a number of publicly available datasets. We first consider two recent metagenomic datasets, one taken from a human gut (approx. 145, 000 reads using Sanger sequencing) [16] and the other from the gut of an obese mouse (approx. 675, 000 reads using 454 sequencing) [17].

Using the mouse gut dataset, we show a discovery rate analysis in Figure 1. From this, we can estimate that doubling the number of sampled read sequences will only lead to the discovery of approximately 50 additional taxa. This result, therefore suggests that this particular metagenome consists of roughly 950 predominant taxa, a large majority of which are already identified using only half of the reads. This example illustrates that the assessment of the species discovery rate per number of reads may be highly beneficial for the design and economy of any project with unknown species composition. Cost savings are likely to be realizable for any project that proves to have a much lower taxonomical diversity than assumed at the outset [28].

In Figure 4, we show a multiple-comparative tree view of the human gut and mouse gut metagenomes, using normalized counts. The analysis is based on a BLASTX comparison of all reads against the NR database. At first glance, there appears to be many nodes at the taxonomical rank of class for which the number of assigned reads differs substantially. However, using the described statistical test, we see that there are only a few statistically significant differences, listed in Table 1.

Because the two datasets were obtained using different sequencing technologies, it may be that some adjustments to the analysis will have to be made to account for the different read-length distributions of multiple data sets. This is ongoing work.

We now briefly discuss the five main differences identified in Figure 4 and Table 1. As expected, Actinobacteria are more dominant in the human gut, manifested through a high abundance of *Bifidobacterium longum*, *B. adolescentis *and *Collinsella aerofaciens ATCC 25986*. All three species are known to be normal inhabitants of the human intestine.

Also, Firmicutes are more dominant in the human gut, mostly in the form of Clostridia, Lactobacillales and Mollicutes. Clostridia and Lactobacillales can live in intestinal tracts of animals and humans, however it is not clear why the levels of abundance differ in the two datasets. The human dataset also contains *Eubacterium dolichum DSM 3991 *whose presence has previously been established by its isolation from the human gut flora. *Mesoplasma florum *is considered a commensal strain in humans and an animal parasite. A striking contrast between the two datasets also seems to be the high abundance of Euryarchaeota/Methanobacteria. As previously reported, the main representative of this group is *Methanobrevibacter smithii*, a well-known archaeal inhabitant of the human gut, see [16,29].

In our experience, the class of Chordata is always problematic in this type of metagenomic analysis. This is most likely due to the high complexity and large sequence space covered by higher eukaryote and especially vertebrate genomes. This is further aggravated by database biases toward model organisms and the problem of false annotation of vertebrate genetic elements.

The amount of hits mapped to Ascomycota was significantly higher in the mouse gut probe, mostly reads assigned to yeast species like *Saccharomyces *and *Candida*. It is well known that these yeast species can be found in caeca of mouse [30] and rat [31]. As stated in [17], the mouse gut probe was extracted from its caecum, whereas the human probe was taken from distal gut.

Interestingly, the proportion of mouse gut reads that exhibit no hits to the NR database is much higher than for the other dataset. This probably reflects the different read lengths produced by the employed sequencing technologies (Sanger for the human gut sample, 454 for the mouse one). An additional potential explanation may be that there is a bias in NR database that favors human endosymbionts and parasites. A basic functional analysis of the mouse dataset can be obtained from the COGs present in the NR database. We show the result of such an analysis in Figure 2.

As a second example, we analyze a set of approx. 140, 000 reads extracted from a soil sample using Sanger sequencing [18] and then compare this to a small subset of approx. 145, 000 reads of the *Global Ocean Survey *dataset, [19] obtained using Sanger sequencing technology. The analysis is based on a BLASTX comparison of all reads against the NR database. In Figure 5 we show a multiple-comparative tree view of the two datasets.

We now briefly discuss some of the main differences summarized in Figure 5 and Table 2. Our analysis reiterates the well-known fact that soil metagenomes are significantly more complex than marine ones. However, this diversity is underrepresented in current reference databases. Therefore, more reads are assigned to the proteobacterial phylum in the marine dataset than in the soil one, in particular *Pseudomonas mendocina ymp*, *Shewanella *(aquatic bacteria), and some unclassified gamma proteobacteria, such as marine gamma proteobacteria HTCC2080, HTCC2143 and EBAC20E09. Differences in the number of reads assigned to Cyanobacteria can be attributed to *Synechococcus *and *Prochlorococcus marinus *which both belong to the most abundant bacterial species in marine surface water [21].

Significantly more reads are assigned to Acidobacteria in the soil dataset, most mapping to *Solibacter usitatus Ellin6076*, a soil bacterium. However, since the Acidobacteria are a very divergent class of taxa, this discrepancy could be due to the low amount of currently sequenced species within this group. The fact that reads hitting Chlorophyta are more present in the marine dataset is due to the number of hits to Prasinophyceae, which are marine algae. The existence of fresh water variants may explain the small number of hits in soil. Reads that match Chloroflexi are found more often in the soil than in the marine dataset, in particular *Herpetosiphon aurantiacus ATCC 23779*, which was originally isolated from a lake in Minnesota, the same state from which the soil sample was taken. The fact that Thermoprotei are favored by the marine sample is due to reads assigned to *Nitrosopumilus maritimus SCM1*, which is a mesophilic (not thermophilic) salt-water bacterium. The groups Oligohymenophorea and Aconoidasida both belong to the phylum Alveolata comprising a very divergent group of unicellular eukaryotes, some of them are capable of photosynthesis. Accordingly, the marine dataset contains significantly more reads of these eukaryotic clades than the soil dataset. Interestingly, most hits within Aconoidasida belong to the taxon *Plasmodium falciparum*, the pathogen of malaria. Since it is known that *P. falciparum *possesses a chloroplast-like organelle which presumably was derived in a common ancestor of Apicomplexa [32], a possible explanation may be that these reads come from a marine species that is closely related to the Aconoidasida but not well represented in the NR database.

In Figure 3 we analyse the microbial attributes content of the soil dataset. Of 564 microbes identified in the dataset, 510 where found among the ≈ 1500 prokaryotes currently listed in the NCBI "Prokaryotic Attributes Table". Somewhat disappointingly, this profile of attributes differs only insignificantly from the one computed for the marine dataset (not shown), most likely due to database biases.

The comparison of the soil and marine datasets can be performed at different levels of the NCBI taxonomy and represented as bar charts, see Figure 6.

## Conclusion

Comparative metagenomics is a fast growing field and novel tools are required to support comparative analysis of multiple metagenomic datasets. In this paper we have discussed a number of new techniques that address this issue. These will all be made available in a new version 2.0 of MEGAN.

We anticipate that a metagenomic project will routinely look at 5–10 different samples, each consisting of 100,000 or more reads. Once the data has been compared against appropriate reference databases, MEGAN 2.0 can be used for fast and user-friendly comparative analyses of datasets of this size, providing graphical support for the publication process.

A number of papers on new metagenome datasets that employ MEGAN as the primary analysis tool are in preparation. Future improvements of the program will include the use of the GO gene ontology [33] to classify functional content and the implementation of more statistical tools for comparing different datasets. MEGAN 2.0 is currently being incorporated into the CAMERA metagenomics web portal [13].

## Availability

The datasets discussed are available at [34]. Installers for common operating systems for MEGAN 2.0 will be available at [20].

## Competing interests

The authors declare that they have no competing interests.

## Authors' contributions

DHH, DCR and SM implemented the comparative methods for MEGAN 2.0. AFA, DCR and SM curated and analyzed the datasets. All authors participated in writing the manuscript. DHH and SCS supervised the study.
